# Supplementary material for: The Importance of Endoplasmic Reticulum Stress as a Novel Antidepressant Drug Target and Its Potential Impact on CNS Disorders
Source: Pharmaceutics. 2022 Apr 12;14(4):846. doi: 10.3390/pharmaceutics14040846 (PMC9032101; doi:10.3390/pharmaceutics14040846)
Supplement: Supplementary file 1 [file pharmaceutics-14-00846-s001.zip › pharmaceutics-1647066-supplementary.pdf]

Studied	a	b	c	d	e	f	g	h	I
**gene**	**TM**	**Ami+TM**	**Esci+TM**	**R-ket+TM**	**S-ket+TM**	**Δ b-a**	**Δ c-a**	**Δ d-a**	**Δ e-a**
ATF4	2.87 ±0.28	3.19 ±0.43	3.03 ±0.35	3.28 ±0.45	3.35 ±0.32				
ATF6	2.50 ±0.31	2.63 ±0.30	2.52 ±0.39	2.58 ±0.30	2.30 ±0.22				
CREB3	2.34 ±0.23	1.59 ±0.13	2.24 ±0.29	2.23 ±0.19	4.01 ±1.27				↑
CREB3L1	1.49 ±0.13	1.47 ±0.25	1.44 ±0.31	2.99 ±0.67	2.50 ±0.45			↑ *	↑
CREB3L4	3.91 ±0.99	3.64 ±1.66	5.50 ±2.89	3.09 ±1.57	6.07 ±1.32		↑		↑
DDIT3	13.75 ±1.08	16.58 ±2.09	14.2 ±1.25	18.31 ±1.88	19.59 ±1.09	↑↑		↑↑ *	↑↑↑ **
EDEM1	3.85 ±0.35	4.08 ±0.63	3.61 ±0.63	5.08 ±0.50	5.55 ±0.56			↑	↑
EIF2AK3	1.78 ±0.17	2.04 ±0.35	1.67 ±0.34	1.95 ±0.23	2.56 ±0.67				
EIF2S1	0.99 ±0.14	0.90 ±0.16	0.74 ±0.21	0.72 ±0.13	0.70 ±0.13				
ERN1	3.61 ±0.37	3.36 ±0.71	3.99 ±1.03	6.37 ±1.59	8.26 ±2.10			↑↑	↑↑↑ *
GABPA	1.14 ±0.10	1.36 ±0.07	1.00 ±0.18	1.15 ±0.18	1.28 ±0.30				
HSPA5	19.36 ±1.26	20.06 ±3.62	19.09 ±3.00	17.54 ±2.25	20.07 ±2.54			↓	
MAP3K4	1.35±0.14	1.60±0.27	1.29±0.24	1.62 ±0.44	1.56 ±0.30				

Table S1. *Effect of tunicamycin (TM) with antidepressants (Ami- amitryptyline, Esci – escitalopram, R-ket - R-ketamine, and S-ket - S-ketamine) and tunicamycin alone in astrocytes with regard to the expression of genes associated with ER stress. To illustrate the difference (Δ) in gene expression of antidepressants the symbols were introduced:* ↑ for 1-2.5 *fold change*; ↑↑ 2.6-4.5 *fold change*; ↑↑↑ > 4.6 *fold change. Data are presented as mean ± SEM and expressed as fold change vs untreated control cells. Statistical significance vs tunicamycin-treated cells is indicated when appropriate; ** $p < 0.01$; *$p < 0.05$.*
